# Molecular Cytogenetic Characterization of Multiple Intrachromosomal Rearrangements in Two Representatives of the Genus *Turdus* (Turdidae, Passeriformes)

**DOI:** 10.1371/journal.pone.0103338

**Published:** 2014-07-24

**Authors:** Rafael Kretschmer, Ricardo José Gunski, Analía Del Valle Garnero, Ivanete de Oliveira Furo, Patricia C. M. O'Brien, Malcolm A. Ferguson-Smith, Edivaldo Herculano Corrêa de Oliveira

**Affiliations:** 1 Programa de Pós Graduação em Ciências Biológicas, Universidade Federal do Pampa, São Gabriel, Rio Grande do Sul, Brazil; 2 Laboratório de Cultura de Tecidos e Citogenética, SAMAM, Instituto Evandro Chagas, Ananindeua, Pará, Brazil; 3 Programa de Pós Graduação em Genética e Biologia Molecular, Universidade Federal do Pará, Belém, Pará, Brazil; 4 Cambridge Resource Centre for Comparative Genomics, Cambridge, United Kingdom; 5 Instiuto de Ciências Exatas e Naturais, Universidade Federal do Pará, Belém, Pará, Brazil; University of Florence, Italy

## Abstract

*Turdus rufiventris* and *Turdus albicollis*, two songbirds belonging to the family Turdidae (Aves, Passeriformes) were studied by C-banding, 18S rDNA, as well as the use of whole chromosome probes derived from *Gallus gallus* (GGA) and *Leucopternis albicollis* (LAL). They showed very similar karyotypes, with 2n = 78 and the same pattern of distribution of heterochromatic blocks and hybridization patterns. However, the analysis of 18/28S rDNA has shown differences in the number of NOR-bearing chromosomes and ribosomal clusters. The hybridization pattern of GGA macrochromosomes was similar to the one found in songbirds studied by Fluorescent *in situ* hybridization, with fission of GGA 1 and GGA 4 chromosomes. In contrast, LAL chromosome paintings revealed a complex pattern of intrachromosomal rearrangements (paracentric and pericentric inversions) on chromosome 2, which corresponds to GGA1q. The first inversion changed the chromosomal morphology and the second and third inversions changed the order of chromosome segments. Karyotype analysis in *Turdus* revealed that this genus has derived characteristics in relation to the putative avian ancestral karyotype, highlighting the importance of using new tools for analysis of chromosomal evolution in birds, such as the probes derived from *L. albicollis*, which make it possible to identify intrachromosomal rearrangements not visible with the use of GGA chromosome painting solely.

## Introduction

Apparently, chromosomal evolution in birds represents an example of evolutionary stability, with karyotypes characterized, in the majority of the cases, by low rates of interchromosomal rearrangements [1]. As an exception to this rule, these rearrangements are common in species with low diploid number, such as the harpy eagle (*Harpia harpyja*), with 2n = 58 and the stone curlew (*Burhinus oedicnemus*), with 2n = 42, which belong to orders Falconiformes and Charadriiformes, respectively [2], [3]. On the other hand, intrachromosomal rearrangements are more frequent, as suggested by the comparison of sequence alignment of entire chromosomes among different species. Recently, such comparisons between chicken, turkey and zebra finch allowed the visualization of many intrachromosomal rearrangements, as well as some interchromosomal ones [4]. Hence, besides differences in lineage-specific gene family expansions and the number of long-terminal-repeat-based retrotransposons, 114 intrachromosomal changes (inversions and translocations) were proposed between chicken and zebra finch, especially in the syntenic groups coresponding to *Gallus gallus* pairs 1 and 2 [5], [6].

The application of comparative chromosome painting using chicken probes has been used efficiently to identify interchromosomal rearrangements, such as fissions and translocations. For each species analyzed, an average of two interchromosomal rearrangements was found, except in more derived karyotype, such as the ones observed in birds of prey (Falconiformes) [7], [8], [9]. Indeed, the chromosome complement of Falconiformes species, which are characterized for presenting a few pairs of microchromosomes, at least 19 to 22 interchromosomal rearrangements have been already described in comparison to the putative ancestral avian karyotype (PAK) [2], [9], [10]. Furthermores, the use of the new developed GGA microchromosome specific probes might increase this average of chromosomal rearrangements, as most of the comparative chromosome painting analyses in birds have applied usually whole chromosome probes covering the macrochromosome GGA pairs 1 to 10 and Z chromosome [11].

Because of these findings, the use of whole chromosome probes derived from a bird of prey should reveal the occurrence of intrachromosomal rearrangements, because individual pairs would correspond to regions within GGA macrochoromosomes. The first reciprocal chromosome painting experiments between chicken and a bird of prey (*Leucopternis albicollis*) confirmed the occurrence of multiple fissions of GGA1, GGA2, GGA3, as well as reinforced the chromosome signature GGA1pter/GGA6 as a synapomorphy shared by four different Buteoninae species, illustrating the importance of the use of comparative chromosome painting in phylogenetic and evolutionary studies [10], [12].

Moreover, other types of probes used in fluorescence *in situ* hybridization (FISH) techniques have permitted the inference of apomorphic or plesiomorphic states of some chromosomal features. For example, the presence of only one pair of chromosomes with 18S/28S rDNA in species of the Cathartidae family (*Gymnogyps californianus*, *Sarcoramphus papa*, *Cathartes aura* and *Cathartes burrovianus*) is considered as a plesiomorphic state of this family [13], [14], since species belonging to the Paleognathae birds (*Pterocnemia pennata*, *Dromaius novaehollandiae*, *Casuarius casuarius*, *Struthio camelus* and *Rhea americana*) also present only one pair with 18S-28S rDNA [15].

Different from Falconiformes, Passeriformes typically show a diploid chromosome number of around 80, with a few exceptions observed in the Tyrannidae family [16]. Comparative painting in this order has revealed the conservation of the majority of chicken macrochromosomes (GGA), except pairs GGA 1 and GGA 4 [7], [8], [17], [18]. Ten species of Passeriformes have had their karyotypes analyzed by chromosome painting so far, comprising representatives of six family, and the data reveal the shared occurrence of a fission of the ancestral GGA 1, resulting in two pairs, probably corresponding to GGA1p and GGA1q [7], [8], [17], [18]. However, this rearrangement does not indicate an apomorphy exclusive to Passeriformes, since the fission of chromosome GGA 1 was also found in species of Strigiformes, Psittaciformes and Falconiformes [2], [7], [9], [10], [19], [20]. On the other hand, *Sylvia atricapilla* shares a derived chromosomal trait with at least three species of different orders – Galliformes, Columbiformes and Anseriformes-, which corresponds to the fusion of PAK4 (GGA4q) and PAK11 (GGA4p) [18], [21].

Following this tendency, species of Turdidae family present a typical avian karyotype, with diploid numbers ranging between 78 and 80 [7], [8], [22], [23]. Little else is known about the chromosomal complement of these species. Only *T*. *merula* and *T*. *iliacus* were studied in more detail by FISH technique [7], [8]. Hence, in order to elucidate the karyotypic evolution in the genus *Turdus*, and confirm intrachromosomal rearrangements suggested by sequence maps of Passeriformes, we characterized the karyotypes of two Neotropical species of *Turdus* (*T. rufiventris* and *T. albicollis*), using classical cytogenetic techniques, as well as comparative chromosome painting with chicken and *Leucopternis albicollis* probes. The results were compared to other species, and a possible sequence of events is proposed for their chromosomal evolution.

## Materials and Methods

### Specimens analyzed

Experiments followed protocols approved by the Ethics Committee on the use of animals (CEUA-Universidade Federal do Pampa) (Permission Number: 026/2012). The specimens were caught in sites which were not privately owned nor protected, and permissions were responsibility of IBAMA (SISBIO 33860-3). Six specimens of *T. rufiventris* and one of *T. albicollis* were collected in São Gabriel and Dom Pedrito cities – Rio Grande do Sul State, Brasil, with mist nets (Table 1).

**Table 1:** Specimen information and number of samples used in this study.

Species	N	Locality/State	2N	Geographical coordinates
*T. rufiventris*	5♂+1♀	São Gabriel, RS	78	30° 20′ 06.56′′ S, 054° 21′ 44.82′′ W
*T. albicollis*	1♂	São Gabriel, RS	78	30° 20′ 06.56′′ S, 054° 21′ 44.82′′ W

### Chromosome preparation

Two protocols were used to obtain chromosome preparations: short-term bone marrow culture and fibroblast culture [24], [25]. The protocols included hypotonic treatment with colchicine (0,05%), treatment with 0.075 M KCl and cell fixation in methanol/acetic acid (3∶1).

### Giemsa staining and C-banding

Karyotype analysis was undertaken using conventionally stained metaphases (Giemsa 5% in 0.07 M phosphate buffer, pH 6.8). Thirty metaphases were analyzed to determinate the diploid number for each species. Afterwards, the chromosome preparations were submitted to C-banding according to Sumner [26].

### Fluorescent *in situ* hybridization

A biotin labelled 18S/28S chicken ribosomal DNA probe was used to detect the location of ribosomal RNA gene clusters. Comparative painting was performed using whole chromosome probes of *Gallus gallus* corresponding to the first 10 pairs and Z chromosomes, and *Leucopternis albicollis* corresponding to pairs homologous to region of GGA1 (LAL 3, 6, 7, 15 and 18), 2 (LAL 2, 4, and 20), 3 (LAL 9, 13, 17 and 26), 4 (LAL 1 and 16), 5 (LAL 5) and 6 (LAL 3) [10]. Both sets were developed by flow cytometry at Cambridge Resource Centre for Comparative Genomics (Cambridge, United Kingdom). Protocols for hybridization, stringency washes and detection were realized as described previously by de Oliveira et al. [10]. Slides were analyzed using Zeiss Axioplan2 fluorescent microscope and Axionvisio 4.8 software (Zeiss, Germany).

## Results

### Karyotype Description

**Figure 1:**
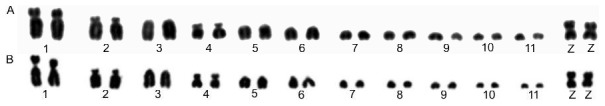
Giemsa-stained partial karyotypes of (A) male Rufous-bellied Thrush (*T. rufiventris*) 2n = 78 and (B) male White-necked Thrush (*T. albicollis*) 2n = 78.

The diploid chromosome numbers were 78 in *T. rufiventris* and *T. albicollis*. In both species, the first and fourth chromosome pairs were submetacentric, the second pair was acrocentric and others were telocentric. The Z chromosome was metacentric in both species and the W chromosome was telocentric in *T. rufiventris* (Fig. 1 A–B).

### C-banding and 18S/28S rDNA probes

**Figure 2:**
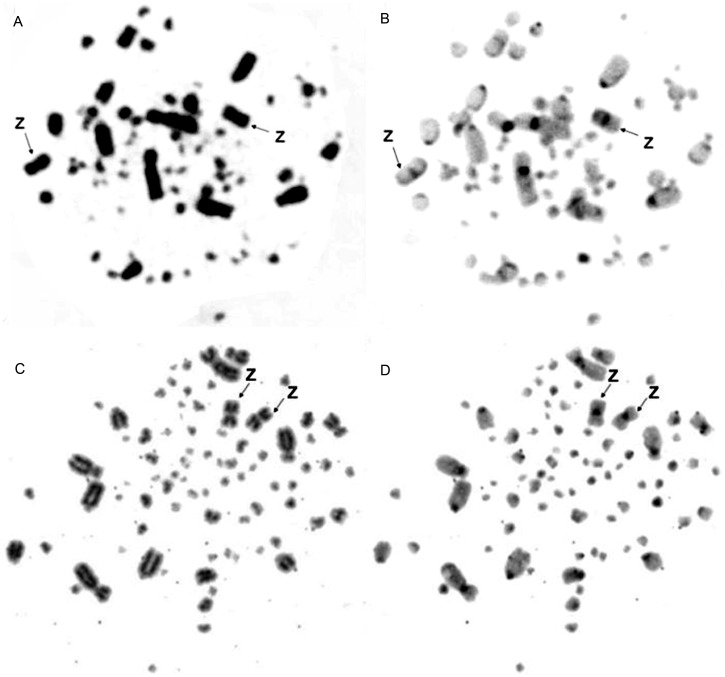
C-banding patterns of: male *T. rufiventris*: Giemsa (A) and C-banding (B); male *T. albicollis*: Giemsa (C) and C-banding (D). The arrows show the sex chromosomes.

**Figure 3:**
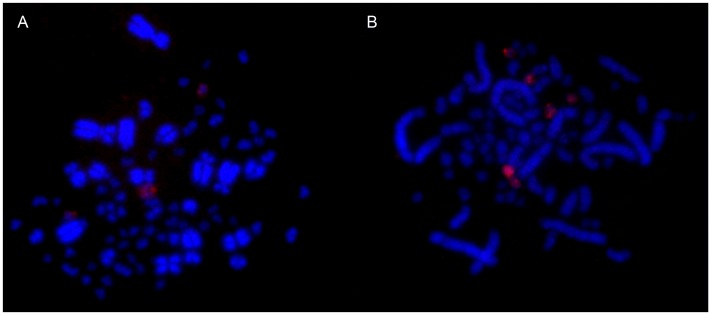
Biotinilated probes localized 18S rDNA loci on two pairs of microchromosomes in *T. albicollis* (A) and in three pairs of microchromosomes in *T. rufiventris* (B).

C-banding analysis revealed the presence of constitutive heterochromatin mainly in the centromeric regions of macrochromosomes and in some microchromosomes in both species. The Z chromosome has centromeric C-bands in both species (Fig. 2 A–D). The 18S/28S rDNA genes were localized on two pairs of microchromosomes in *T. albicollis* and in three pairs of microchromosomes in *T. rufiventris* (Fig. 3).

### Chromosome painting

Both sets of chromosome specific probes (GGA and LAL) delivered reproducible results when hybridized to metaphases of *T. albicollis* and *T. rufiventris*. The chromosome paintings derived from LAL produced brighter signals and less background when compared to the results of GGA probes.

**Figure 4:**
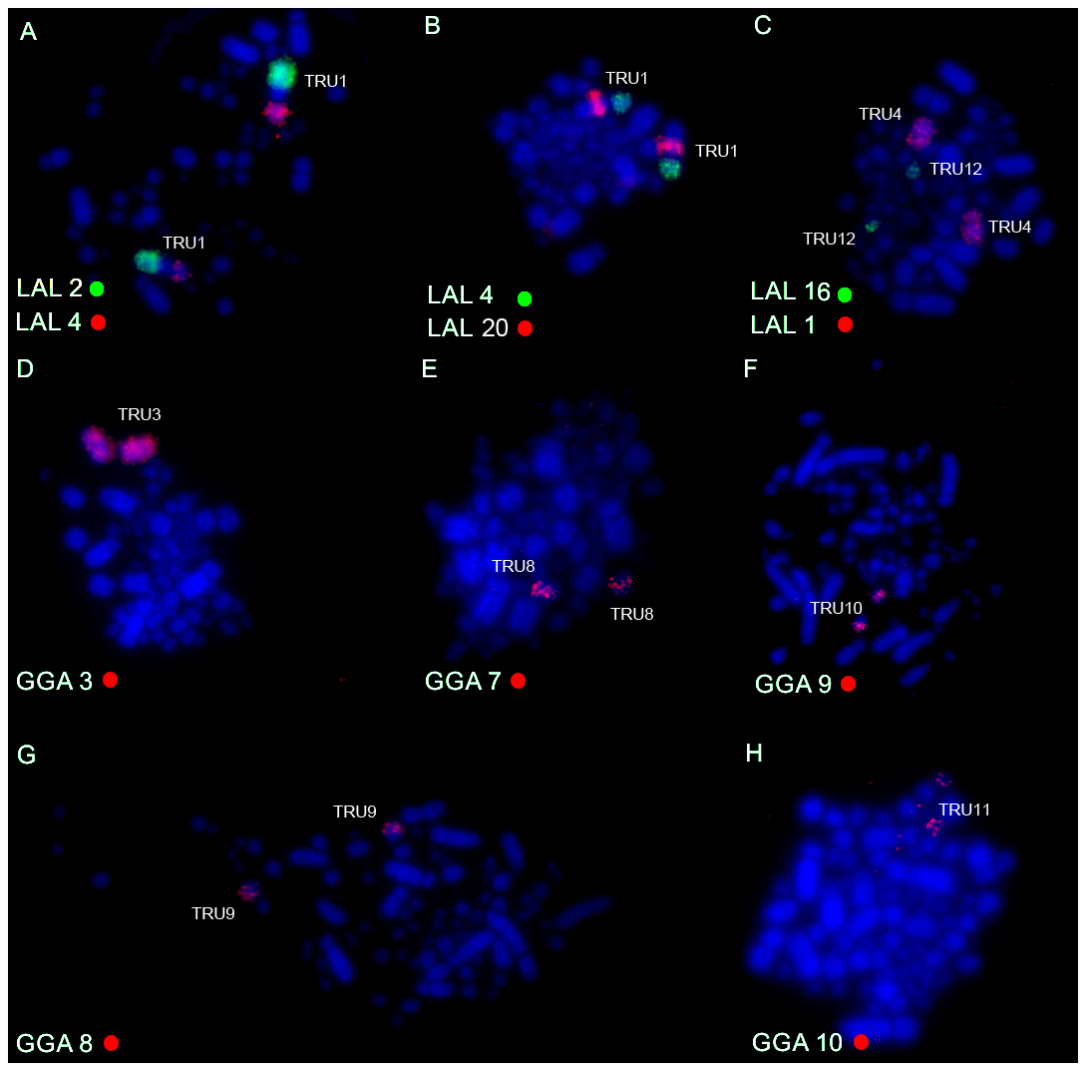
Representative FISH experiments with chromosome painting probes of *Leucopternis albicollis* (LAL) (A–C) and *Gallus gallus* (GGA) (D–H) hybridized onto *Turdus rufiventris* metaphases. The chromosome probes used are indicated on the left bottom, in green (fluoroscein labelled) or red (biotin-cy3 labelled).

**Figure 5:**
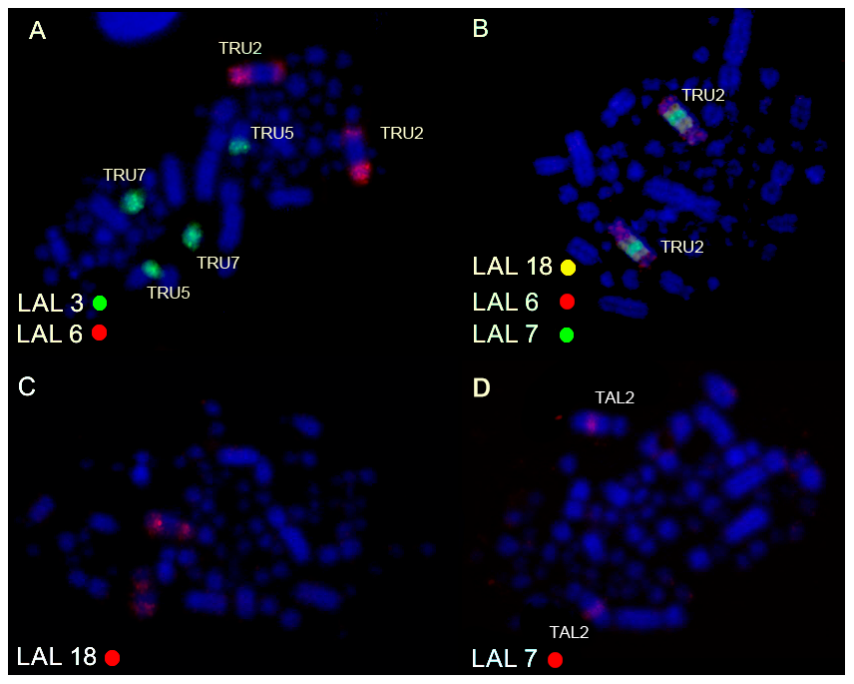
Whole chromosome probe derived from *L. albicollis* which are involved in the fission of ancestral GGA 1 chromosome and inversions on chromosome 2 in *T. rufiventris* (A–B) and *T. albicollis* (C,D). The chromosome probes used are indicated on the bottom left, in green (fluoroscein labelled), red (biotin- or anti-digoxigenin-cy3 labelled) or yellow (biotin-cy3 labelled).

**Figure 6:**
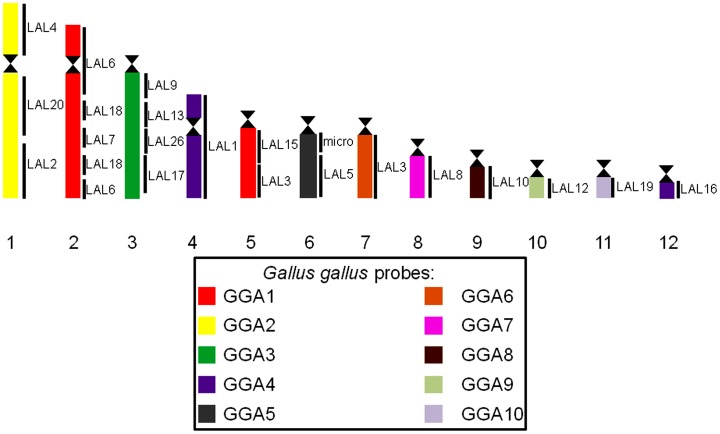
Homologous chromosomal segments in chicken (*Gallus gallus*), *L. albicollis* and *Turdus* macrochromosomes as detected by fluorescence *in situ* hybridization (FISH) using chicken and *L. albicollis* whole chromosome paints.

Chicken macrochromosome painting (10 autosomal pairs and the Z chromosome) on the two *Turdus* species showed a high degree of conservation of macrochromosomes, producing 13 distinct signals in both species. The only exceptions were chromosomes GGA 1 and GGA 4, which hybridized to two chromosomes pairs in both species, corresponding to pairs 2 and 5 (GGA1q and GGA1p, respectively), and pairs 4 and 12 (GGA4q and GGA4p, respectively) (Fig. 4 A–H; Fig. 5 A–D). Pairs 2, 3, 5, 6, 7, 8, 9, 10 and chromosome Z of GGA corresponded to *Turdus* 1, 3, 6, 7, 8, 9, 10, 11 and Z, respectively. *L. albicollis* probes, in a total of 15 different chromosome paintings, produced 16 signals in both species of Turdus: only the synteny corresponding to LAL 3 was not conserved, producing signals in two different pairs (5 and 7) of *Turdus*. Differently of chicken probes, which revealed an apparent conservation in *Turdus*, *L. albicollis* probes corresponding to GGA1q produced a different sequence when compared to the results observed in *Gallus gallus*, evidencing the occurrence of intrachromosomal rearrangements (Fig. 5 A–D). In some metaphases, cross-hybridization was produced in pairs 2 and 3 of *Turdus* by LAL 20 (homologous to a segment of GGA2). Figure 6 shows the homology between the GGA and LAL probes in *T*. *rufiventris* and *T*. *albicollis*.

## Discussion

### Karyotypic characterization

Of the sixty-six species of the genus *Turdus*, ten have already been karyotyped by conventional analysis. These analyses showed high chromosomal similarity and the presence of the typical karyotype for the class Aves, with high diploid number, few pairs of macrochromosomes and many microchromosomes pairs. The two species analyzed herein showed similar karyotypes, and no differences in diploid number or morphology of macrochromosomes were observed. Our results corroborate the findings of Giannoni et al. [23], with *T. rufiventris* and *T. albicollis* presenting 2n = 78, with a metacentric Z and a telocentric W.

C-banded segments were confined mainly to the centromeric regions of macrochromosomes and of some microchromosomes in both species. This pattern was found in other species of Passeriformes, as for example, in *Ramphocelus carbo* and *Tangara cayana* [27]. The Z chromosome showed positive C-bands in the centromere. This distribution of blocks of constitutive heterocromatin is typical for avian karyotypes.

18S/28S rDNA probes hybridized onto two and three pairs of microchromosomes in *T. albicollis* and *T. rufiventris*, respectively. These results can be considered as a derived characteristic (apomorphic) in relation to the putative avian ancestral karyotype. This hypothesis is based in the fact that more basal species (Paleognathae) such as *Pterocnemia pennata*, *Dromaius novaehollandiae*, *Casuarius casuarius*, *Struthio camelus* and *Rhea americana* have only one pair with 18S/28S rDNA [15]. A possible explanation for this difference in number of NOR-bearing chromosomes is the amplification of ribosomal genes after a translocation event [28]. Chromosomal rearrangements in *T. albicollis* and *T. rufiventris*

**Figure 7:**
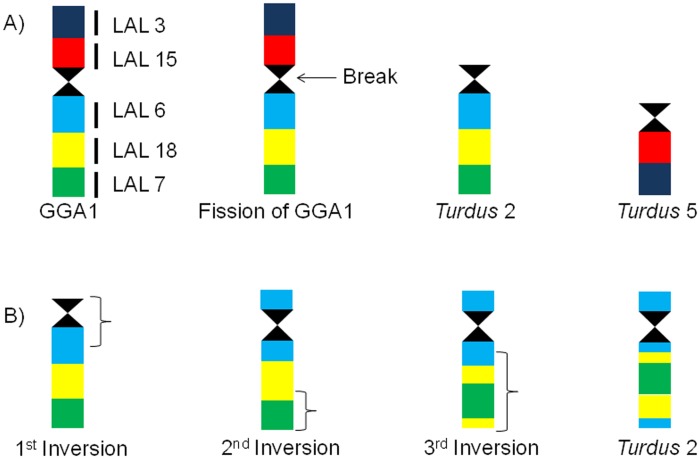
Schematic representation illustrating the origin of chromosomes 2 (GGA1q) and 5 (GGA1p) in species of the genus *Turdus*, and intrachromosomal rearrangements in chromosomes 2, considering chromosome GGA1 as the ancestral state. The first event was a centric fission, which originated *Turdus* 2 and 5 (A). Posteriorly, *Turdus* 2 had three inversions, the first was pericentric, and the second and third were paracentric ones (B).

The results of GGA and LAL chromosome painting in two species of genus *Turdus* confirmed the conservation of macrochromosomal syntenies, with the exception of GGA 1 and GGA 4, which were separated by fission into two chromosome pairs. GGA 1 fission seems to be shared by different species of Passeriformes, according to the available results of FISH [7], [8], [17], [18]. Apart from this fission, no intrachromosomal rearrangement was identified by the use of chicken probes. However, an interesting result was observed from LAL chromosome painting: after the centric fission of GGA1, different inversions occurred in chromosome 2 of *T. rufiventris* and *T. albicollis*, which corresponds to GGA1q (Fig. 7).

If we assume that the sequence of LAL probes in *Gallus*, also observed in the turkey vulture (*Cathartes aura*) [29], is an ancestral character (although analyses in other species of birds must be done do confirm this assumption), the first inversion, pericentric, must have involved only the segment corresponding to LAL 6, changing the telocentric morphology to acrocentric. Posteriorly, two paracentric inversions involved only the long arm of the *Turdus* 2. The breakpoints are not coincident with those observed in *L. albicollis*, as indicated by the multiple signals produced by some probes. These inversions were proposed from the comparison of sequence alignments of chicken and zebra finch (*Taeniopygia guttata*), the first songbird belonging to the large avian order Passeriformes which has had its genome sequenced [6]. Probably, these inversions are present in *T*. *merula* [7] and *T*. *iliacus* [8] but, however, were not detected by the use of chicken macrochromosome probes solely.

Considering that chromosomal rearrangements play a key role in speciation and genome evolution, including also influence in social behavior in some species [11], [30], whole chromosome probes of species in which macrochromosomes are fissioned in multiple chromosome pairs provide an important tool for analysis of these phenoma in birds. For instance, our results illustrate well the advantage of using *L. albicollis* probes, considering that in this species, some chicken macrochromosomes are involved in several fission events, the most extreme example being GGA 1 chromosome, which is homologous to five distinct pairs of *L. albicollis* [10]. Hence, with the hybridization of these five chromosomes, it was possible to detect intrachromosomal rearrangements, not observed with chicken probes. Because *Gallus gallus* and *Cathartes aura*, two phylogenetically distant species, show the same order of these segments identified by LAL probes [29], we propose that the intrachromosomal rearrangements observe in *Turdus* are derived characteristics when compared to the putative avian ancestral karyotype. In addition, it is also important to consider possible effects of these intrachromosomal rearrangements, which seem to occur frequently in the genomic evolution in birds [4]. For instance, the observation that chromosomal polymorphism due to an inversion on chromosome 2 of the white-throated sparrow (*Zonotrichia albicollis*) influenced the variation in social behavior and plumage, shows that chromosomal inversions can have unique and established genetic characteristics influencing profoundly the evolutionary process [30].

The study reported in the present paper demonstrate that analyses with LAL probes in other species of birds are important and necessary to verify when the intrachromosomal rearrangements observed in the segment homologous to GGA1 occurred during the avian karyotype evolution or if they are exclusive to the genus *Turdus*. Also, studies focusing on the effects of these rearrangements could clarify the real effects of inversions in the genomic evolution of birds.
